# Correction: Heat, hurricanes, and health: Effects of natural disturbances on angling effort

**DOI:** 10.1371/journal.pone.0295716

**Published:** 2023-12-07

**Authors:** The PLOS ONE Staff

## Notice of Republication

This article was republished on November 22, 2023, to correct the error in the affiliation that was introduced during the typesetting process. The publisher apologizes for the error. Please download this article again to view the correct version. The originally published, uncorrected article and the republished, corrected articles are provided here for reference.Reference

## Supporting information

S1 File Originally published, uncorrected article. (PDF)

S2 File Republished, corrected article. (PDF)
