# Supplementary material for: Heat, hurricanes, and health: Effects of natural disturbances on angling effort
Source: PLoS One. 2023 Sep 8;18(9):e0291126. doi: 10.1371/journal.pone.0291126 (PMC10490919; doi:10.1371/journal.pone.0291126)
Supplement: S1 Table — (DOCX) [file pone.0291126.s001.docx]

**Table S1.** Weekly effort distribution (%) as determined by LDWF between 2015 and 2021.

| **Year** | **Mon** | **Tues** | **Wed** | **Thu** | **Fri** | **Sat** | **Sun** |
| --- | --- | --- | --- | --- | --- | --- | --- |
| 2015 | 8 | 8 | 9 | 10 | 18 | 32 | 15 |
| 2016 | 8 | 7 | 8 | 10 | 20 | 32 | 16 |
| 2017 | 8 | 8 | 7 | 10 | 19 | 32 | 17 |
| 2018 | 7 | 7 | 7 | 10 | 21 | 34 | 14 |
| 2019 | 7 | 7 | 7 | 11 | 21 | 33 | 16 |
| 2020 | 7 | 7 | 7 | 10 | 21 | 33 | 16 |
| 2021 | 6 | 7 | 8 | 11 | 21 | 31 | 15 |
| Mean | 7.3 | 7.3 | 7.6 | 10.3 | 20.1 | 32.4 | 15.6 |
