# Supplementary material for: Correction: Heat, hurricanes, and health: Effects of natural disturbances on angling effort
Source: PLoS One. 2023 Dec 7;18(12):e0295716. doi: 10.1371/journal.pone.0295716 (PMC10703275; doi:10.1371/journal.pone.0295716)
Supplement: S1 File — (PDF) [file pone.0295716.s001.pdf]

PLOS ONE

RESEARCH ARTICLE

Heat, hurricanes, and health: Effects of natural disturbances on angling effort

Stephen R. Midway*, Paul W. Miller

Department of Oceanography and Coastal Sciences, Louisiana State University, Baton Rouge, Los Angeles, United States of America

* smidway@lsu.edu

Abstract

Recreational angling is a very popular outdoor activity that is weather-dependent, although investigations of this relationship are rare. This study used weekly fishing effort (2015–2021) estimates throughout coastal Louisiana to understand how effort changed in response to weather conditions. Although we found evidence for some effect of all the weather variables, temperature reported the greatest number of monthly effects, along with an overall declining effect throughout the year. We also examined how tropical storms and hurricanes reduce fishing effort, but that effort recovers rapidly after the storm. Finally, we examined fishing effort during the first year of the pandemic (2020) compared to previous years and found some monthly increases exceeding 100% of normal effort. Understanding angler motivations remains an important part of fishery management, and in a future with changes to weather, hurricanes, and global health crises, we can now know more about how environmental factors change angling effort.

Check for updates

OPEN ACCESS

Citation: Midway SR, Miller PW (2023) Heat, hurricanes, and health: Effects of natural disturbances on angling effort. PLoS ONE 18(9): e0291126. https://doi.org/10.1371/journal.pone.0291126

Editor: Mariya Gubareva, ISEG Lisbon School of Economics and Management, PORTUGAL

Received: October 14, 2022

Accepted: August 22, 2023

Published: September 8, 2023

Copyright: © 2023 Midway, Miller. This is an open access article distributed under the terms of the Creative Commons Attribution License, which permits unrestricted use, distribution, and reproduction in any medium, provided the original author and source are credited.

Data Availability Statement: All data were from publicly available sources that are described in the manuscript.

Funding: The author(s) received no specific funding for this work.

Competing interests: The authors have declared that no competing interests exist.

Introduction

The influence of weather and season on human outdoor behavior is well accepted; studies have even documented the correlation between favorable weather and employees reporting sick [1]. However, the generality that warmer and dryer weather leads to more outdoor activity may be too simple. Across the globe, weather and seasons are constantly changing—subsequently presenting conditions to different populations that have varying tolerances for temperature [2], precipitation, and wind [3], among other conditions. Specific and local understanding behind outdoor recreation decisions is important not only from a human behavior standpoint, but because estimating the number of human users can help resource managers understand and anticipate environmental use and resource exploitation.

Efforts to understand the effect of weather on outdoor recreation have been ongoing for many years; for example, Verbos and Brownlee [4] developed the weather dependency framework, which is a model to understand the effect of weather on outdoor recreation. Meanwhile, the Weather Channel® produces activity-specific indices to communicate the favorable combinations of both biotic and abiotic factors for multiple forms of recreation, such as the Golf Index, the Ski Index, and the Fishing Index [5]. Other studies have focused on specific weather variables. Paudyal et al. [6] reported the greatest number of daily visitors to the Florida

National Scenic Trail to occur between 16–22˚C (among other findings), and Finger and Lehmann [7] documented temperature and rainfall to be significant factors in driving visitors to lidos (public beaches) in Switzerland. On a seasonal level, disparities in weather patterns between the mid-latitudes and tropical regions are a huge driver of the Caribbean tourism season [8]. Despite apparent relationships—such as warmer temperatures lead to increased recreation—it is important to recognize that weather variables may have non-linear effects across larger variable ranges. In a review of season and weather on physical activity, Tucker and Gilliland [9] found that although summer was often the season of greatest physical activity, this was not true for Galveston, Texas because of extreme heat [10].

Recreational fishing is a popular outdoor activity across the globe and extremely popular in the United States. An estimated 49 million anglers [11] spend $50 billion USD in the US annually [12], based on recent analyses. The vast majority of US anglers are licensed and many target fish species under some form of fishery management. Therefore, an accurate understanding of angler effort and predictors of angler effort can be valuable to the fishery management process. However, such predictors can assume a wide variety of forms that operate on widely varying timescales. For instance, short-term weather conditions can affect angling decisions on daily scales (i.e., precipitation, wind) as well as seasonal scales (i.e., cold in winter versus warmth in summer). Simultaneously, discrete and high-impact climate events may disrupt angling effort for months after they occur by damaging the fishing infrastructure (e.g., boat launches, marinas) that supports angler effort. Meanwhile, other natural events, such as infectious disease outbreaks (i.e., COVID-19), can alter angling effort for years depending on public health policies. The multi-scale influence of these natural disturbances and their ability to predict subsequent angling effort are largely unknown within the fishery management community.

As far back as 1979, researchers have investigated how weather (in this case temperature and rainfall) was significantly related to fishing effort [13]. One study that did not even directly measure weather or effort learned from angler surveys that weather significantly influenced effort [14]. A handful of recent studies from across the globe have also attempted to quantify the effect of temperature, precipitation, and wind on angler efforts. Griffin et al. [15] not only found effects of weather variables on angler effort in Australia but that there were different magnitudes of effects based on different types of fishing, specifically whether anglers were fishing from a boat or from the shore. Hunt et al. [16] reported that maximum temperature and precipitation both had negative effects on angler activity in Ontario Canada, and Labriji et al. [17] reported on wind speed as a factor influencing fishing effort (although this was a single-species commercial fishery in Africa). Dundas and von Haefen [18] provide what is perhaps the most comprehensive and regionally appropriate analysis (when compared to our study), in which they used Marine Recreational Information Program (MRIP; [19]) data to document an inverted U-shape temperature response with reduced angling at temperatures less than 40˚F and greater than 95˚F. This temperature profile largely aligns with other reports of outdoor activity preferring 82˚F [20].

While tropical cyclones (TCs; referring to both tropical storms and hurricanes) have been shown to pose persistent environmental consequences beyond their acute wind and surge threats [21], only recently have we begun to learn about the varying effects that tropical storms have on coastal fish populations. Tropical storms have been documented to effect both individual species populations (e.g. Atlantic croaker *Micropogonias undulatus* in the Chesapeake Bay, USA [22]) and fish assemblages—although recent work suggests minimal effects of TCs on fish (in New Jersey, USA [23]; in Florida, USA [24]; and in Louisiana, USA [25]). While a wide range of effects on fish abundance and behavior may not be directly connected to angler effort, it is well known that many anglers have an understanding of how fish respond to their

environment, and as a result anglers may fish more or less based on their perceptions of relative abundance and location of target fish species.

Early work has reported a wide range of COVID-19 pandemic effects on anglers (henceforth referred to as the *pandemic*). A survey of recreational anglers from 15 countries reported an overall reduction in marine recreational fishing [26]. Yet in the US, studies thus far have suggested the opposite—that recreational angling effort substantially increased as a result of the pandemic. MRIP data has shown the average number of fishing trips increasing by 44% from 2019 to 2020, with the greatest increases in the US Gulf of Mexico and Atlantic regions [27], and an independent survey of anglers in 10 US states found that, although pandemic effects were heterogeneous across states, there was a net increase in angling effort during the first three months of the pandemic (March–May 2020; [28]).

The goal of this study is to better understand the effects of weather, TCs, and the COVID-19 pandemic on marine recreational anglers in the northern Gulf of Mexico. The northern Gulf of Mexico presents an attractive study system for a number of reasons. First, the state of Louisiana records angler effort at the weekly scale, which provides far more temporal resolution than most other large scale effort surveys. (For comparison the federal MRIP survey provides angler effort at the time scale of two months.) Louisiana also provides spatial resolution to their angler effort data, whereby inshore and offshore effort can be attributed to regions within the state. Coastal Louisiana's subtropical climate also means that, while overall temperatures may be considered mild, sub-freezing temperatures can still occur in the winter, and summers experience extreme heat [29]. The northern Gulf of Mexico also experiences regular TCs, and this regularity permits us to investigate TCs with some measure of replication that would not be afforded in other areas [30]. Finally, Louisiana experienced some of the highest rates of COVID cases relative to the rest of the US [31], which leaves no doubt that fishing effort for much of 2020 was taking place during the strong influence of the pandemic (compared to other regions of the US that did not see as many COVID cases or did not have strong pandemic waves in 2020). The objectives of this study are to 1) quantify the effect of ambient weather variables on angling effort, 2) evaluate the response of fishing effort after TCs, 3) and to document fishing effort in 2020 relative to non-pandemic years.

Methods

Data preparation

Fishing effort data. Fishing effort data was acquired from the Louisiana Department of Wildlife and Fisheries (LDWF) as part of their LA Creel recreational fishery monitoring program [32]. The LA Creel program is a combined weekly access-point survey (boat launches, marinas, etc.) to estimate catch (harvest) along with weekly phone and email surveys to licensed anglers to estimate fishing effort, which is measured in single-day, single-angler trips [32]. Although Louisiana previously participated in the federal MRIP program (and its predecessor, Marine Recreational Fisheries Statistics Survey, MRFSS), the National Oceanographic and Atmospheric Administration approved LA Creel for use in 2018. LA Creel provides estimates for both offshore and basin-specific inshore locations on a weekly basis, which makes it one the most high-resolution long-term creel monitoring programs in the US.

Weekly effort estimates are available starting in 2015 (with weeks defined as starting on Monday and ending on the following Sunday) and are available spatially across five inshore coastal study areas shown in Fig 1a: Pontchartrain (1), Barataria (3), Terrebonne/Timbalier (5), Vermilion/Teche (6), and Calcasieu (7) as well as a single aggregated offshore area. Effort within coastal study areas is further split into charter or private, and for this analysis we only consider private data, which represents individual recreational anglers. To accommodate the

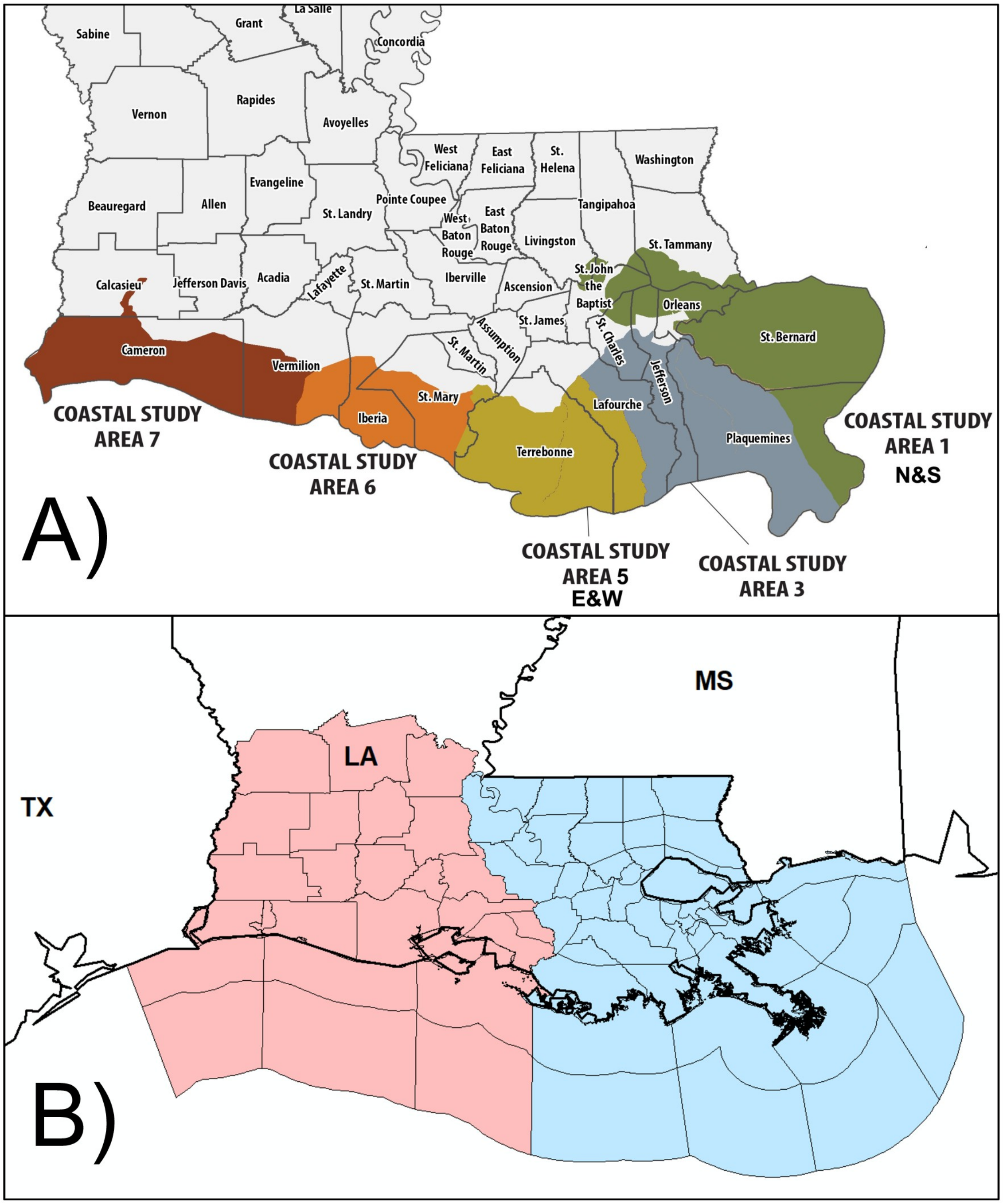

Fig 1. (A) LDWF coastal study areas (Credit: wlf.louisiana.gov). (B) National Weather Service forecast zones within southern Louisiana and adjacent coastal waters. Red zones are administered by the Lake Charles NWSFO whereas blue zones are under the jurisdiction of the Slidell/New Orleans NWSFO. Onshore zones generally correspond to parish political boundaries, except in some coastal parishes where the parish is divided into an inner, land-centric forecast zones and an outer, coastal zone.

https://doi.org/10.1371/journal.pone.0291126.g001

mobility of anglers between coastal study areas (e.g., someone who lives in Pontchartrain and assesses weather conditions at his or her home but fishes in Barataria where their effort is registered in LA Creel), we aggregated Pontchartrain, Barataria, and Terrebonne/Timbalier into the *east* region and Vermilion/Teche, and Calcasieu into the *west* region Recent travel

estimates of (n = 1,136) coastal Louisiana recreational anglers reported an average one-way distance for a fishing trip to be 86 miles [33], which would keep most anglers fishing within the region that they live.

Regional delineations were also guided by the bifurcation of southern Louisiana between two National Weather Service (NWS) forecasting offices (NWSFOs; Fig 1b). In Louisiana, two NWSFOs issue weather forecasts and warnings for the coastal parishes, one based in Lake Charles, LA, and the other in Slidell, LA. Although individual coastal study areas may be of interest, the NWSFO boundary perfectly aligns with the border between Terrebonne/Timbalier and Vermilion/Teche coastal study areas (Fig 1), providing a clean separation of the effort data by region. Because the NWS weather warnings are later leveraged as a proxy for weather conditions (see next section), the aggregation of coastal study areas along NWSFO boundary was a logical parallelism for the analysis. All offshore effort was removed from the analysis because the regional designation is ambiguous; i.e., offshore landings could not be attributed to the east or west.

Of particular importance is the fact that, although effort estimates are produced by week, effort varies depending on day of the week. LDWF estimates about 70% of effort taking place on the weekend—as defined by Friday, Saturday, and Sunday—with the other 30% of effort on weekdays (Monday, Tuesday, Wednesday, and Thursday; [32]; S1 Table). Although our analysis does not look at time at scales finer than a week, this within-week variability is not only important for understanding effort but was also included in how we effort-weighted some of the weather variables (see next section).

Weather data. Meteorological conditions were characterized using 2-m temperature, precipitation, and 10-m wind speed from the ERA5 atmospheric reanalysis [34], a 0.25-degree gridded global dataset. Gridded hourly meteorological data covering all of southern Louisiana and adjacent coastal waters were collected for the period 2015–2021, coincident with the period of record for the LA Creel survey. Because weather conditions, particularly wind and precipitation, may not be consistent across the entire Louisiana coast at any given time, weather conditions were summarized for the regions shown in Fig 1b. For instance, an active thunderstorm day near New Orleans, may reduce effort over the southeast region, while southwest Louisiana may experience little precipitation and sunny weather conditions.

Each hourly variable was averaged over the regions depicted in Fig 1b, yielding two values per variable per hour. Regional means were then summarized on a daily basis as follows: maximum temperature (˚C), total precipitation (mm), and maximum wind speed (m/sec). Other characterizations of daily weather conditions were also explored (e.g., mean temperature, maximum wind speed, heat index); however, all like-variable-derived metrics (i.e., mean temperature vs maximum temperature; mean wind speed vs maximum wind speed) were strongly colinear, and only one of each was retained.

Because the LA Creel survey is collected weekly, the daily meteorological conditions were aggregated to match the temporal resolution of the effort data. Rather than employing a traditional mean, this analysis opted for a weighted average based on the historical distribution of fishing effort according to the LA Creel survey (S1 Table). For instance, weekends are the most heavily fished days of the week, so an uncomfortably cold Saturday would have a disproportionate effect on weekly effort compared to an uncomfortably cold Tuesday. Weekly variable aggregations resulted in each region having 364 weekly effort values (52 weeks over 7 years) paired with corresponding effort-weighted maximum temperature, mean precipitation, and maximum wind speed.

Because many anglers, especially those fishing from watercraft, closely monitor National Weather Service forecasts for sea conditions, weekly counts of marine advisory issuance were catalogued alongside the meteorological conditions. Archived watch, warning, and advisory

Table 1. Total number of NWS advisories and warnings issued for forecast zones within each of the CWAs constituting the two study regions between 2015–2021.

NWSFO (region)	Small craft advisory	Tropical Storm Warning	Hurricane Warning
Slidell (east)	4100	154	68
Lake Charles (west)	2137	78	26

https://doi.org/10.1371/journal.pone.0291126.t001

products for the offshore zones in Fig 1b were retrieved from the publicly accessible Iowa Mesonet database (https://mesonet.agron.iastate.edu/vtec/). Three types of marine forecast products are considered, all of which indicate poor fishing conditions: Small craft advisories, tropical storm warnings, and hurricane warnings. Though the NWS issues multiple advisories and warnings that might portend poor coastal fishing conditions (e.g., special marine warnings, gale warnings and dense fog warnings), such products were either issued infrequently or designed to be issued reactively after a decision to go fishing has likely been made. The total number of small craft advisories issued by each NWSFO over its forecast zones during the LA Creel survey week were summed alongside the effort data (Table 1). Large small craft advisory counts reflect a combination of persistent rough seas during the week as well as the areal expanse of the afflicted waters. Weekly aggregation was repeated for both tropical storm and hurricane warnings (Table 1). NWS warning and advisory patterns can vary across issuing offices (Table 1), further necessitating the regional stratification of the effort analysis.

According to the Slidell NWSFO webpage (https://www.weather.gov/lix/wwa_criteria), small craft advisories are issued whenever sustained winds of 21–33 kt, frequent gusts to 21–33 kt, or waves $\geq$7 feet are expected within 36 hours. Meanwhile, tropical storm (hurricane) warnings indicate that sustained winds of 34–63 kt ($\geq$64 kt) associated with a TC are expected within 36 hours. Both tropical storm and hurricane warnings indicate that high winds may be accompanied by storm surge, coastal flooding, and/or river flooding. In some cases, the 36-hour lead time for these advisories and warnings caused a slight disconnect between the LA Creel week when the warning was issued and the week when the effort was impacted. These infrequent instances were manually resolved on a case-by-case basis.

Data analysis

Effect of weather on fishing effort. Although there are many weather variables that could conceivably be considered, we identified four variables of interest: *precipitation*, *wind*, *temperature*, and *small craft advisory frequency* (Fig 2). Each of these variables represents a different potential effect on angler effort. Precipitation can discourage fishing by creating uncomfortable and unsafe conditions for the angler along with equipment challenges. Maximum wind speed represented our hypothesis for wind, under the idea that anglers are more sensitive to maximum winds, which would generate the roughest fishing conditions, instead of minimum or mean wind speeds. Maximum temperature was also used under the same logic as wind—the maximum temperature is what would likely motivate angler behavior over other measures of temperature, especially in a warm climate like coastal Louisiana where extreme heat is common in several months of the year (and more common than extreme cold). Additionally, anglers likely base their assessment of future fishing conditions as informed by television or internet-based forecasts that typically communicate heat via the daily high temperature. Finally, the weekly total count of small craft advisories represents a more holistic and official designation of wind that also communicates specific information about expected sea conditions. Based on expert opinion, we thought this advisory was the most relevant to anglers

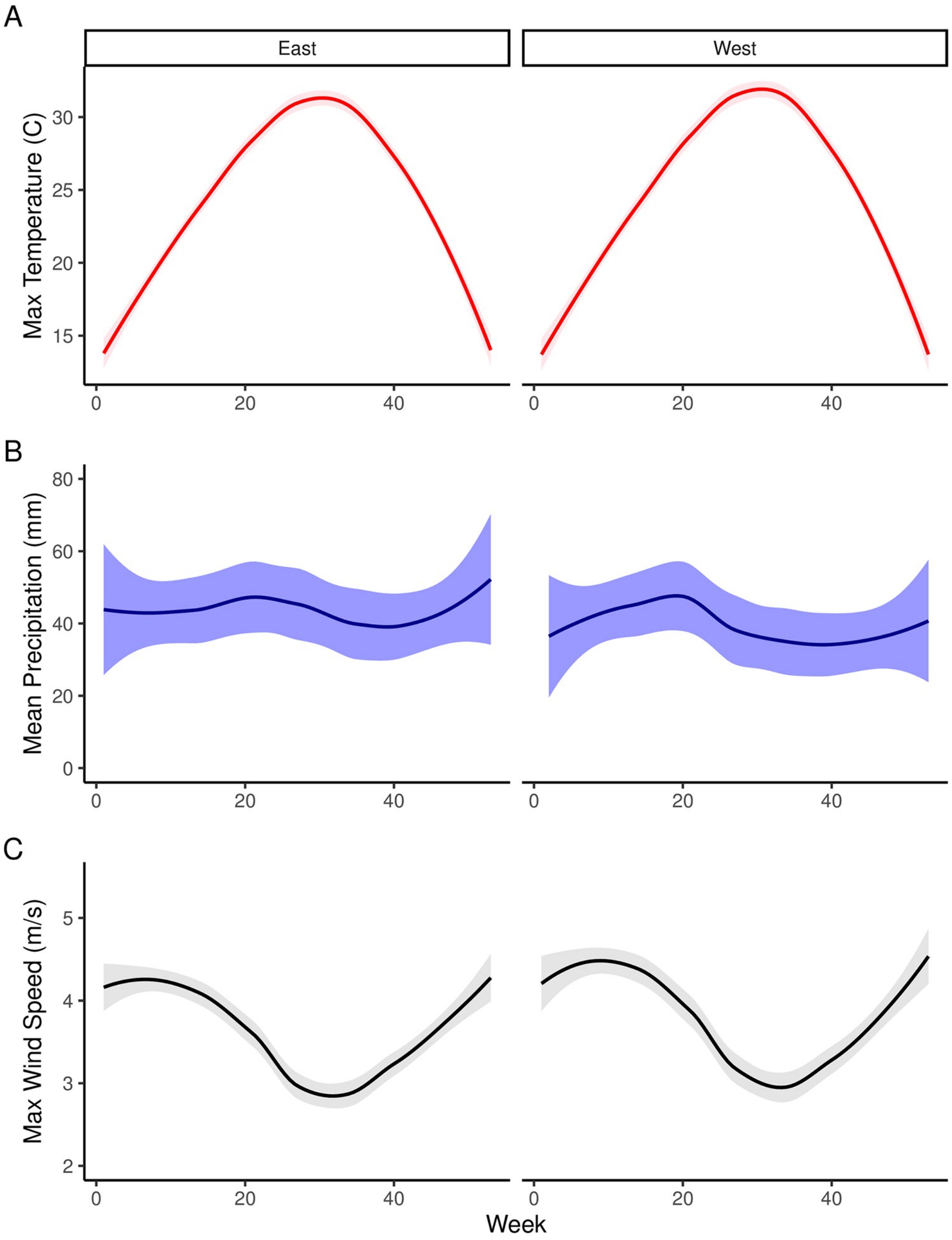

Fig 2. Annual cycles of effort-weighted meteorological variables for the 2015–2021 study period for each coastal region. Dark lines represent the 7-yr locally weighted scatterplot smooth (LOESS) and shaded regions correspond to the 95% confidence interval.

https://doi.org/10.1371/journal.pone.0291126.g002

because of the connection to boating and consequently communicated unique information that was not redundant to the effort-weighted maximum wind speed.

Before using any of the four predictors, we ran a Pearson correlations on all pairwise combinations of predictors and found the greatest correlation was -0.57 (between temperature and small craft advisory), which was below conventional thresholds for concerns about multicollinearity. Finally, because the objective of this analysis was to look at the possible effect of different types of weather, we did not want other confounding effects included in the data. We removed weeks with hurricane warnings and tropical storm warnings (which were analyzed

separately), weeks in which Memorial Day, Independence Day, and Labor Day holidays fell (due to the well-known increase in effort associated with these long, warm-weather weekends), and all of 2020—in order to remove any potential effect of the pandemic (which was also separately analyzed).

To model the potential effects of weather variables on angling effort, we used a Bayesian hierarchical model. We included a random effect of *month* in the model because we wanted to allow for the potential magnitude and direction of the effect to change throughout the year. As examples, the effect of wind may have a greater magnitude in cooler months than in warmer months, or the effect of temperature may have a positive direction during cooler months and negative effect during warmer months (Fig 2). The general form of the hierarchical model is as follows:

$$y_i = \alpha_j + \beta_{j1} \times x_{i1} + \beta_{j2} \times x_{i2} + \beta_{j3} \times x_{i3} + \beta_{j4} \times x_{i4} + \epsilon_i \tag{1}$$

$$\epsilon_{ij} \sim N(0, \sigma^2) \tag{2}$$

$$\begin{pmatrix} \beta_{j1} \\ \beta_{j2} \\ \beta_{j3} \\ \beta_{j4} \end{pmatrix} \sim MVN(\boldsymbol{\mu}, \boldsymbol{\Sigma}) \tag{3}$$

$$\boldsymbol{\mu} = (\hat{\beta}_{j1}, \hat{\beta}_{j2}, \hat{\beta}_{j3}, \hat{\beta}_{j4}) \tag{4}$$

Where y_i is the log-transformed effort for week i and α_j is a random intercept for j = 12 months. β terms represent the four model coefficients on the four predictors, x_{1-4}, with subscripts j for monthly random effects and i for weekly observations, as appropriate, and following the order of 1 for *precipitation*, 2 for *temperature*, 3 for *wind*, and 4 for small craft advisory. ϵ_i is the residual error associated with week i (Eq 2). All four predictors were standardized by mean centering and dividing by one standard deviation [35], which both improved model convergence and allows for direct comparisons of the predictor effect sizes. The spatially varying model parameters were assumed to come from a multivariate normal (MVN) distribution (Eq 3), where $\boldsymbol{\mu}$ and $\boldsymbol{\Sigma}$ are the population mean and variance-covariance matrix, respectively. As such, $\hat{\beta}_{j1}, \hat{\beta}_{j2}, \hat{\beta}_{j3}, \hat{\beta}_{j4}$ describe the population-average across all months, using all data. Prior probabilities on all parameters were diffuse. We used diffuse normal priors for $\hat{\beta}_{j1}, \hat{\beta}_{j2}, \hat{\beta}_{j3}, \hat{\beta}_{j4}$ and modeled $\boldsymbol{\Sigma}$ using the scaled inverse-Wishart distribution [35].

To model the potential for month-specific effects on the weather-effort relationship, Eq 3 can be modified as follows:

$$\begin{pmatrix} \beta_{j1} \\ \beta_{j2} \\ \beta_{j3} \\ \beta_{j4} \end{pmatrix} = \begin{pmatrix} \mu_{\beta 1} \\ \gamma_0^{\beta 2} + \gamma_1^{\beta 2} \times z_{month} \\ \mu_{\beta 3} \\ \mu_{\beta 4} \end{pmatrix}. \tag{5}$$

Eq 5 allows for the predictor of *month* to model variation in the monthly change in regression slopes for the coefficient of temperature, β_{j2}. In this relationship, $\gamma_0^{\beta 2}$ represents the intercept for the effect of *month* and $\gamma_1^{\beta 2}$ represents the slope (effect) of *month*, indicted by the

variable, z_{month}. Diffuse normal priors were used for both $\gamma^{\beta 2}$ parameters. Any varying slope parameter in the first level (Eq 1) is a candidate for modeling in this second level of the model; however, we only had a hypothesis for *temperature* and not for other effects, and as such limited our second level investigation to temperature. Keeping the effects of *precipitation*, *wind*, and small craft advisory varying by month does, however, allow us to understand if direction or magnitude of these effects varies by month. Temperature was the only variable for which we expected a predictable change across months; for example, an increase in temperature in cooler months might increase angling effort whereas an increase in temperature in warmer months might decrease angling effort. Note that before deciding on a simple linear regression to test for the effect of month, we evaluated other functional forms. Quadratic, negative exponential, and hyperbolic function were all evaluated in Eq 5, yet they all performed relatively poorly, which is likely due to the fact that there are only 12 points to model in Eq 5. The regression line (from Eq 5) was able to model these 12 points whereas other functions either overfit the data or did not fit at all due to limited contrast in z_{month}.

The model for weather (Eqs 1–5) was run twice: once for the east region and once for the west region. For each model, we ran three parallel Markov chains beginning each chain with different values. From a total of 300,000 samples from the posterior distribution the first 30,000 samples of each chain were discarded then we retained every other sample for a total of 105,000 samples used to characterize the posterior distributions. We assessed convergence for all parameters both visually (trace plots and density plots of posterior distributions), as well as with the Brooks-Gelman-Rubin statistic, $\hat{R}$, with values <1.1 indicating convergence. Analyses were run using JAGS [36] (from the jagsUI package), run from within R [37].

Effect of tropical cyclones on fishing effort. Weeks with ≥ 1 tropical storm or hurricane warning were qualitatively compared against the International Best Track Archive for Climate Stewardship [38], a global database of TC tracks and intensities, to determine if the TC directly impacted Louisiana, and if so, which region was primarily affected. This process was necessary to avoid analyzing the angling effort response of weak TCs that, though they may have met the technical criteria for a tropical storm warning in an offshore zone, passed through the central Gulf of Mexico and posed little practical disruption to nearshore coastal activity. Additionally, this vetting process identified the coastal region that was most impacted by the storm and where changes in angling effort were most likely to occur. In total, 12 TCs from 2015–2021 directly impacted at least one region of Louisiana. To determine the duration of a TC's impact on fishing activity, we wanted to model the effort in the weeks following each TC, under the thinking that TCs and weaker hurricanes would require little recovery time, whereas major hurricanes would see effort continue to increase (i.e., recover) several weeks after landfall. To estimate the effect of TCs on fishing effort we subset the effort data to only include weeks during or after landfall for the 12 TCs. A total of five weeks of effort was included for each TC, where week 1 included the day of landfall. (There were a few instances where landfall occurred early in week 2, though the associated tropical storm/hurricane warning was issued in week 1. Although in these rare cases no TC made landfall in week 1, the impending landfall would be expected to effect angler effort and as such, those weeks were kept).

A Bayesian hierarchical model was also used for this analysis whereby Eq 1. was modified to be a varying intercepts and varying slopes simple linear regression. Weekly effort was again the response variable and *Relative week to landfall* (1–5) was the continuous predictor. Intercepts and slopes varied with the random effect of specific TC. The varying slopes were modeled with a simple linear regression against TC severity at landfall, with 0 indicating a tropical storm and 1–5 following the Saffir-Simpson hurricane wind scale at the time of landfall extracted from IBTRaCS. This second level regression allowed for the possibility to capture the

hypothesized relationship between greater slope estimates corresponding with more severe TCs. In other words, we were testing for the effect that more severe TCs will have both a greater decrease in angling effort at the time of landfall and a slower recovery back to pre-TS levels.

Effect of COVID-19 pandemic on fishing effort. Recreational angling effort in coastal Louisiana was widely reported to be higher than typical effort during the pandemic. Therefore, we did not want to simply test this expectation, but sought to quantify the monthly difference in effort during 2020 compared to all other pre-pandemic years (2015–2019). Note that for this analysis we excluded all of 2021, which in exploratory analyses appeared more like pre-pandemic fishing effort, but could still be considered part of the pandemic and therefore unclear as to how much 2021 was a pandemic year, a non-pandemic year, or some type of new post-pandemic level of effort. Additionally, while there was no strong reason to expect different pandemic effects on the eastern or western regions of the state, we opted to continue with the east and west regions for the pandemic analyses for consistency throughout our study. Additionally, lack of differences between regions could be interpreted as evidence for a single state-wide effect of the pandemic.

Results

Effect of weather on fishing effort

Between two regions and four weather variables in each region we examined a total of 96 potential monthly effects of weather (Fig 3). Small craft advisories appeared to have the least effect on angler effort; no months in the West region ever showed an effect of small craft advisories and only January in the East region showed a significant effect. Maximum wind speed was also largely a non-factor in determining angling effort, with the exception of January and May in the East region and January and March in the West region—all months that showed a significantly negative effect of maximum wind on angler effort. Precipitation was a significant monthly effect for four months of the year in the East region and three months of the year in the West region, and although some of the months differed, the significant months tended to occur in the late winter and again in the late summer. Finally, as expected we saw both significant effects of temperature across months while also documenting an overall decrease in the effect of temperature over the calendar year. Significant temperature effects were exclusively positive and associated with increased fishing effort in the winter, spring, and November in the East region but only in January through March in the West region. September in the West region was the only month that found a negative effect of temperature on angling effort, but the magnitude of the effect was not significant. The second level regression on the effect of month on the slope of temperature found the monthly effect to be negative for both the East and West regions; however, effect was not significant (at the 5% level).

Effect of tropical cyclones on fishing effort

We examined a total of 12 TCs: six tropical storms, two Category-1 hurricanes, one Category-2 hurricane, one Category-3 hurricane, and two Category-4 hurricanes. All 12 TCs were fit for individual relationships between the recovery of fishing effort based on the relative week from landfall. Eleven of 12 TCs had a non-significant slope of effort recovery in the five weeks after landfall (Fig 4), based on effort from the region that experienced the primary storm impact. Hurricane Ida was the only storm in which a significant increase in effort was detected, although several other storms showed positive slopes (that were not significant at the 95% level).

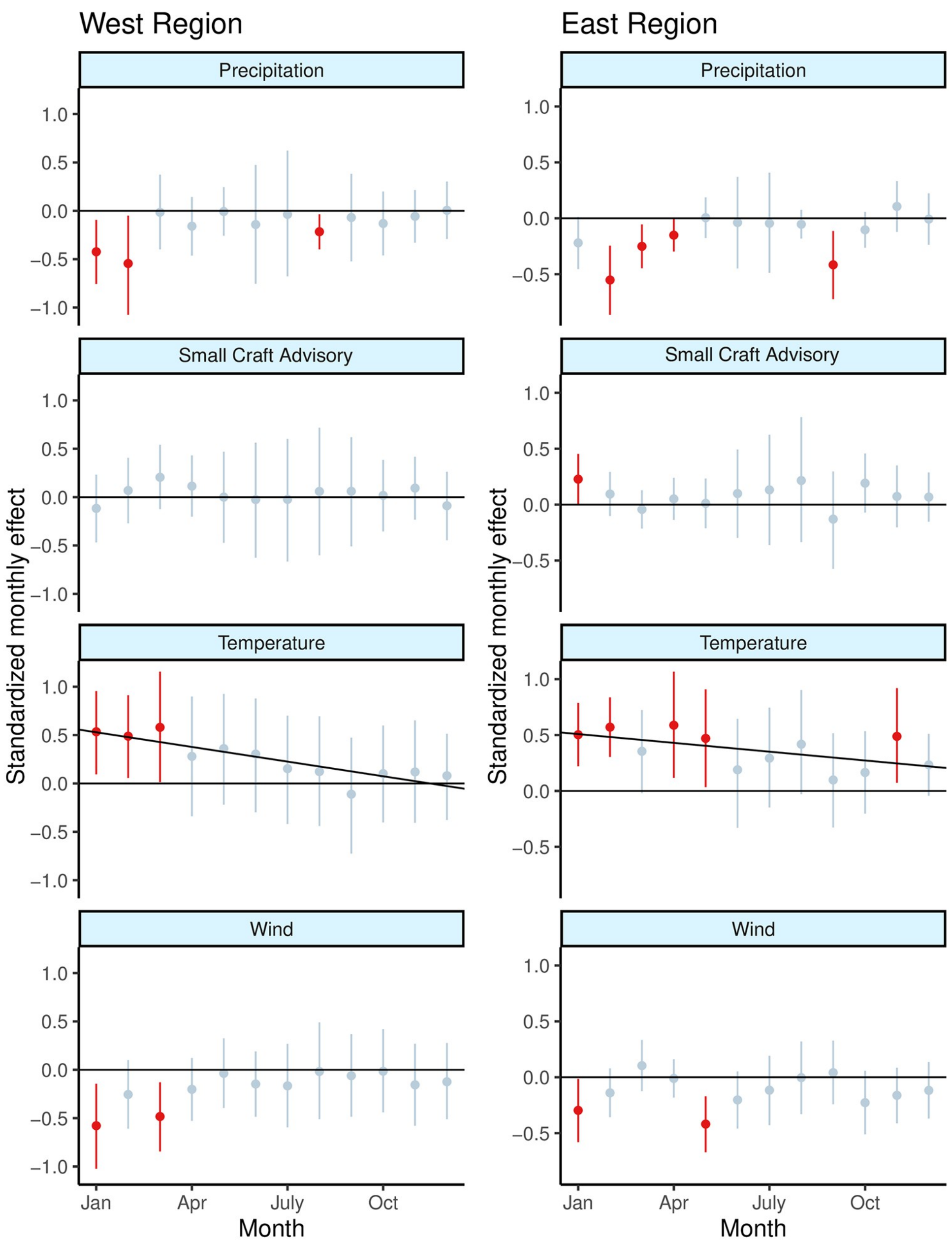

Fig 3. Standardized monthly effect estimates by region for precipitation, small craft advisories, temperature, and maximum wind speed. In each of the eight panels the point represents the estimated mean monthly effect and the intersecting line segment represents the 95% credible interval. Points and lines in red indicate monthly estimates that are not credibly 0, while light blue points and lines indicate estimates that with 95% credible intervals overlapping 0. The black line in both temperature panels represents the level 2 regression on the effect of temperature throughout the calendar year.

https://doi.org/10.1371/journal.pone.0291126.g003

Effect of COVID-19 pandemic on fishing effort

Effort increases in 2020 were clear when compared to the five years prior to the pandemic (2015–2019). January and February 2020 were consistent with typical effort from earlier years (Fig 5), which would be expected given that a global pandemic and local effects in Louisiana were not felt until March. Effort for March, April, and May were substantially higher in 2020

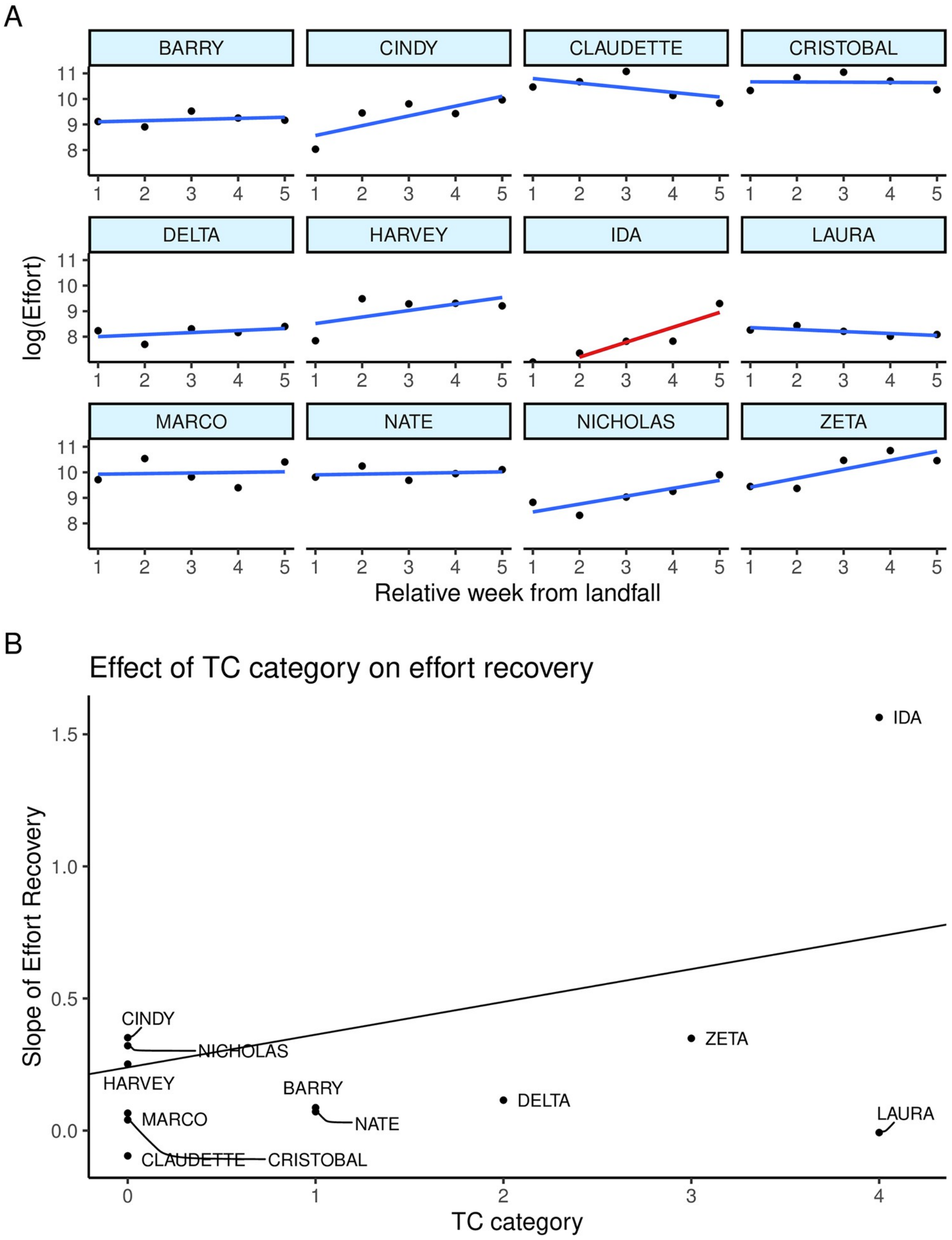

Fig 4. A) Angling effort in the weeks after tropical cyclone landfall. Each panel represents an individual storm that made landfall in Louisiana during our study. Black dots represent effort estimates (on a log scale) in the region corresponding to landfall. The gray lines represent the linear regression fit to the points, with non-significant regression lines in blue and one significant regression line in red. B) Overall slope of recovery effort regressed against tropical cyclone severity. The coefficient estimates of the individual storm recovery regression lines (shown in panel A) are represented by black dots. Estimates are plotted by the category of the storm, with zero representing a tropical storm and 1–4 representing hurricane strength. The black line is the regression of the 12 effort slopes against storm category.

https://doi.org/10.1371/journal.pone.0291126.g004

than in any previous year; effort in the East region for March and April was close to 75% higher than the same months in pre-pandemic years, while effort in the West region was around 150% higher for the same months. May effort was also high, but June and July effort in both regions was remarkably consistent with pre-pandemic levels. August saw another

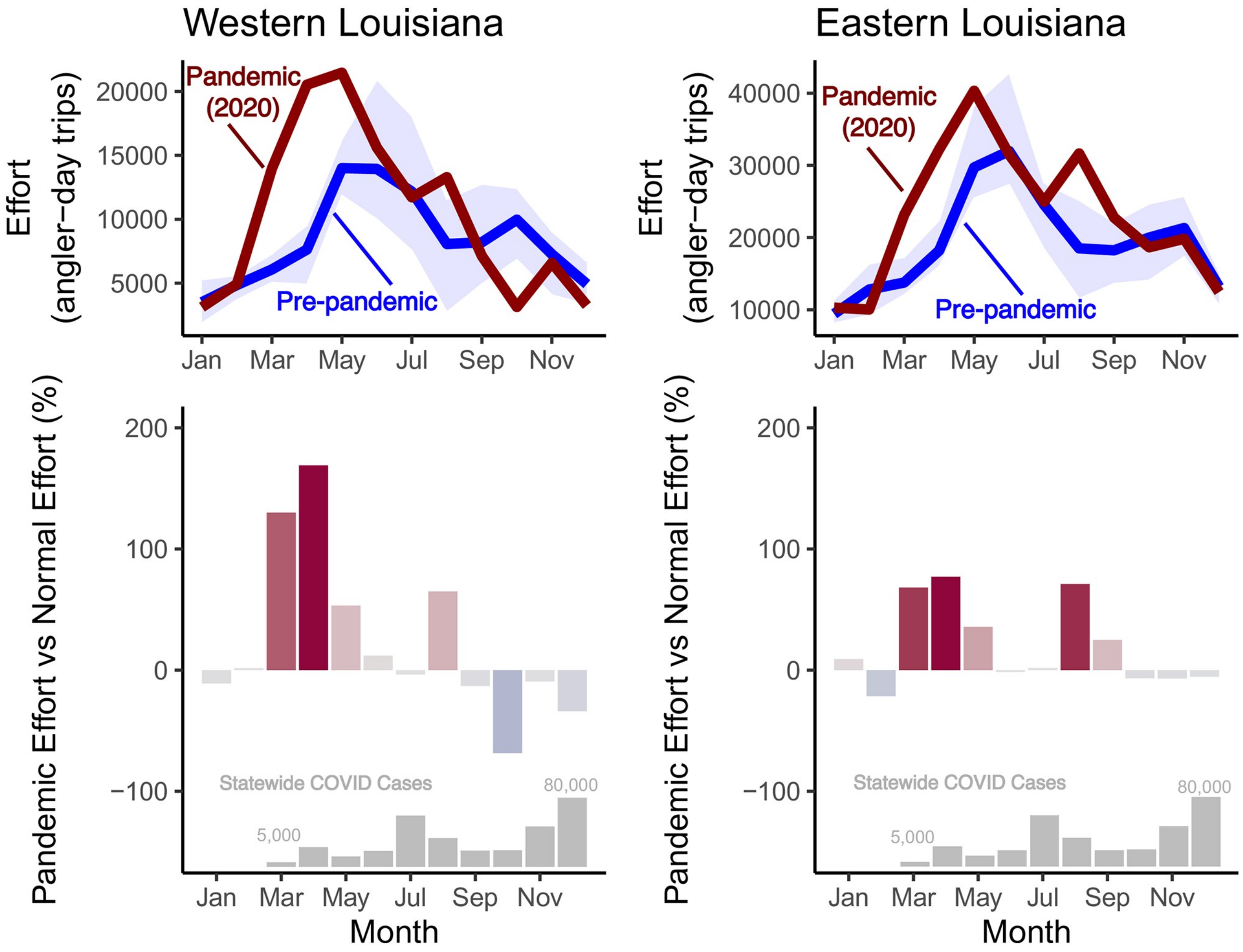

Fig 5. Angling effort in relation to the COVID-19 pandemic. The top two panels show monthly angling effort estimates (by region) during the pandemic year (2020) compared to pre-pandemic angling effort. The dark red line representing 2020 effort does not have any uncertainty because each month is a single estimate, while the pre-pandemic monthly estimates are represented by the blue line with the shaded blue polygon representing the range of effort estimates. The lower two panels show monthly pandemic effort as a percentage of normal (pre-pandemic) effort. Bars with red shading indicate greater pandemic effort than normal effort while blue bars indicate greater normal effort than pandemic effort (with the intensity of color simply representing the magnitude of the difference). At the bottom of the lower panels, for reference, is an inset of monthly COVID cases across all Louisiana (not broken down by region).

https://doi.org/10.1371/journal.pone.0291126.g005

substantial increase in effort, but the remainder of the year in both regions returned to pre-pandemic levels (with the exception of October in the West region, which was substantially lower than pre-pandemic). With a few exceptions, it appears that the pandemic increased recreational angling effort in Louisiana with little regional effect.

Discussion

For many anglers the decision to go fishing involves an increasing amount of information. Brief, impromptu fishing trips close to a residence likely involve fewer risks and therefore fewer decisions; however, many anglers are willing to drive long distances and prepare boats and other equipment in order to fish for many hours or days. Because fishing is an exclusively outdoor activity weather is intuitively a primary consideration informing decisions to fish.

As expected, temperature was often an important driver of fishing effort. Across all of Louisiana we saw strong positive effects of temperature in winter and spring months, which is

easily interpreted as warmer days in cooler months creating conditions that get more people out fishing. This effect of temperature disappeared in the warmer months of the year. Summer and fall in Louisiana—the hottest times of year—saw no significantly positive effect of temperature. This may be attributed to low variability in temperature during summer and early fall compared to the cool season. For instance, the standard deviation for effort-weighted mean temperature in June and July (both regions inclusive) was 1.1˚C and 1.8˚C, respectively. In contrast, the analogous values for January and February were 3.3˚C and 3.6˚C. Because the effort-weighted means were standardized during the data preparation, a standardized value of 1 in January reflects 3x the temperature increase compared to the mean as it does in June. While a warmer-than-average such week in January may motivate recreational anglers to go fishing, the same standardized temperature value in June reflects a relatively negligible change to weather conditions that is unlikely to influence angler behavior.

During most of the summer and fall months the effect of temperature was still slightly positive, which may be explained as warmer days being sunny and less likely to experience precipitation and strong wind. For instance, in the East region, the coefficient of determination (R^2) between temperature and wind speed is 0.001 (p = 0.69) for weeks when the effort-weighted mean temperature was $<$25˚C. However, when the effort-weighted mean was $\geq$25˚C, temperature and wind were negatively correlated with an R^2 of 0.22 ($p<0.001$). This means the warmest weeks in the dataset were generally accompanied by the friendliest wind conditions, perhaps offsetting the undesirable heat. And although it was not a significant effect it was interesting to see that September in the western region of the state did report a negative effect of temperature, suggestive of the idea that at a certain temperature threshold fishing effort does decrease. This may partially be attributed to the accumulated effect of "heat fatigue" throughout the summer. An angler that might have reflexively braved exceptionally warm temperatures to go fishing earlier in the summer may be more willing to abstain after several months of fishing in such conditions. Additionally, an offset in water temperature may be at play, whereby cooler water temperatures (during warm air temperature days) in early summer might allow better fishing while warm water and warm air temperatures in late summer could be less desirable.

Somewhat surprisingly, small craft advisories, precipitation, and wind were not strong indicators of a decrease in fishing effort. Although these three variables were not strongly correlated, it is very possible that they have an interactive effect that informs the decision to go fishing. For example, extreme wind or extreme precipitation alone may stop a fishing trip, but so might moderate wind and light precipitation when they are both present. Future research may consider more closely examining the effects of combinations of meteorological factors on angling effort. Additionally, the small craft advisories were not effort-weighted in the same manner as the other variables, allowing small craft advisories on low-effort days to carry equal weight. Lastly, all three of these variables are more relevant to boat fishing, whereas temperature likely influences all fishing modes (e.g., shore and boat). Thus, the responses to small craft advisories, wind, and precipitation may be dampened due to shore anglers' indifference to poor sea conditions indicated by elevated small craft advisories, wind, and precipitation.

Though the relationship between TCs, fish, and fishing has been studied previously, most studies have tended focus on the post-TC movement of fish populations, livelihoods, and economic impacts on communities. To our knowledge, this is the first study to consider the response of recreational angling to tropical storms and hurricanes. The data we analyzed largely supported our hypothesis that the rate of recovery of recreational angling effort increased with the severity of the TC. Despite this general support we also learned that most storms showed little effect of effort recovery, suggesting that outside of major hurricanes, recreational fishing on the Louisiana coast is resilient to TCs. More severe storms, such as

Hurricanes Zeta and Ida, showed the pattern we expected of an increase in effort with relative week after landfall. We very much expected this same effect from Hurricane Laura, another major hurricane that landed in Louisiana; however, Laura was so devastating that our five-week recovery period was far too short to capture the true recovery time because of the severe damage to fishing infrastructure (boat launches, marinas, etc.). Many residents in the West region were out of power for months and in certain locations infrastructure needed (or remains) to be entirely rebuilt. In addition to these logistical barriers for the region's anglers, persistent power outages would also impede the ability of the LaCreel effort program to call and sample licensed anglers. This historic storm had effects beyond what coastal Louisiana typically experiences and likely accounts for the reason we did not see any slope of recovery of fishing effort.

Another possible limitation of our TC analysis is the spatial scale. Although the East and West regions of Louisiana are generally representative of weather and also meaningful spatial scales for anglers, sometimes a hurricane's landfall can be extremely localized. For instance, Hurricane Ida (2021) made landfall near Port Fourchon, LA, as a Category-4 storm. Nearby Grand Isle, LA, an active coastal fishing community, was devastated by Hurricane Ida. According to the Coastal Emergency Risks Assessment web tool (https://cera.coastalrisk.live/), estimated storm surge exceeded two meters across most along the barrier island community. In contrast, storm surge in Cocodrie, LA, approximately 60 km west of Grand Isle, experience an estimated storm surge of less than 0.3 m. Additionally, protected marinas along the north shore of Lake Pontchartrain experienced much milder surges on the order of 0.5 m, which would have allowed them to reopen with relative swiftness. However, all three of these areas fall within the East region, confounding the interpretation of post-TC recovery. While some communities are severely battered, broader regional fishing infrastructure may remain relatively intact allowing anglers to continue fishing from alternative locations and boat launches.

The effect of the COVID-19 pandemic on recreational angling in Louisiana was generally consistent across the state. The largest increases in fishing effort occurred from March through May, which was the very start of the pandemic. Although emergency "stay at home" orders required non-essential workers to shelter in place, outdoor activities were exempt from these so-called "lockdowns" in Louisiana (https://gov.louisiana.gov/assets/Proclamations/2020/JBE-33-2020.pdf), and it is clear that recreational angling experienced an unprecedented boom as people had time away from work and increases in discretionary time that overlapped with some of the most favorable weather months of the year. The strength of this effect is perhaps clearest in March and April 2020 in western Louisiana during which fishing effort was around 150% above normal effort. Interestingly, June and July of 2020 were near-normal effort months when compared to pre-pandemic effort, and it could be that anglers who had just fished a lot in the spring were decreasing their effort during the warmer months, or were getting back to work after stay-at-home orders were expiring or not being enforced. August 2020 reported large effort increases similar to those in the spring. It is hard to know exactly what was motivating the short-term increase in August, but it could have been related to the second COVID wave that was again causing people to cancel vacations and identify safe outdoor activities that include fishing. Alternatively, Louisiana instituted a statewide mask mandate on July 11, 2020, that may have prompted some anglers to pursue outdoor activities that where masking was not required (https://gov.louisiana.gov/assets/Proclamations/2020/modified/89-JBE-2020-State-of-Emergency-COVID-19-Face-masking-order.pdf). The remainder of 2020 was relatively normal in terms of fishing effort with the exception of October in western Louisiana, which likely includes the long-term effect of Hurricane Laura's damage to roads, marinas, and other infrastructure needed to support recreational fishing. Recent work around the globe has

found the effect of COVID on recreational angling to be very heterogenous with many places showing dramatic decreases in effort [39], and in comparison, Louisiana appears to be a place that was extreme in its change (increase) in fishing effort in 2020.

More robust conclusions regarding the weather-effort relationship were limited by the design of the LaCreel program. Although the catch and effort estimates from the LaCreel program are considered to be among the best in the US, they are still estimates and at the weekly scale could be prone to missing or misrepresenting the true amount of effort taking place across the coast. A second limitation is the resolution of our temporal scale. Decisions to go fishing generally take place at the scale of a day, and it is also very common for weather to change day-to-day. So, although the best unit of study would have been day, we were limited to investigating weather and effort at the scale of week, which in some cases could mute stronger daily patterns. Despite these very real limitations to LaCreel, we still think the LaCreel weekly effort data is very robust and appropriate for this analysis, nor are we aware of any better recreational angling data set that was available.

Conclusion

Successful resource management requires understanding the factors that influence stakeholder participation and extraction of resources, and as management evolves, angler motivation and choice are likely to increase in recreational fishing research. Given this expectation, scientists and managers need an improved understanding of effects outside of the number of licenses or size of the fish population. Our study identifies and quantifies important environmental conditions, both contemporaneous and antecedent, that are associated with elevated fishing effort. Understanding ambient weather, discrete TC events, and public health ordinances not only help us to explain recent and current patterns and fishing effort, but also help us anticipate the future. Based on the relationships we have documented between angler effort and temperature it is possible to consider what recreational angling looks like under various scenarios of climate change. Increases in effort also have impacts on revenue from license sales, use of coastal infrastructure, and support for enforcement. We might also expect the effects of climate change to be heterogeneous; for example, warmer summers in Louisiana may lead to decreases in effort, while warmer winters may lead to increases in effort. These background and subtle long term effort changes can be contrasted to the acute changes we document from the pandemic. It is clear that Louisiana anglers viewed the pandemic—even from the very start—as an opportunity to increase the time they spend fishing. The acute and strong positive effect of the pandemic not only underscores the importance of recreational angling to the state of Louisiana, but also suggests that in future public health crises—and possibly other crises—we might expect similar increases in recreational fishing.

Supporting information

S1 Table. Weekly effort distribution (%) as determined by LDWF between 2015 and 2021.
(DOCX)

Acknowledgments

We thank Kevin Bland and others at the Louisiana Department of Wildlife and Fisheries for use of the LaCreel data, along with all those who help curate and make accessible meteorological data. Bobby Kirk provided early ideas toward our analysis, and Anna B. Kaz assisted with identifying COVID case data.

Author Contributions

Conceptualization: Stephen R. Midway, Paul W. Miller.

Formal analysis: Stephen R. Midway, Paul W. Miller.

Methodology: Stephen R. Midway, Paul W. Miller.

Visualization: Stephen R. Midway, Paul W. Miller.

Writing – original draft: Stephen R. Midway, Paul W. Miller.

Writing – review & editing: Stephen R. Midway, Paul W. Miller.

References

1. Shi J, Skuterud M. Gone Fishing! Reported Sickness Absenteeism and the Weather. Economic Inquiry. 2015; 53(1):388–405. https://doi.org/10.1111/ecin.12109
2. Wilson L, Black D, Veitch C. Heatwaves and the elderly—The role of the GP in reducing morbidity. Aust Fam Physician. 2011; 40(8):637–40. Epub 2011/08/05. PMID: 21814665.
3. Williams CA, Miller PW, Black AW, Knox JA. Throwing Caution to the Wind: National Weather Service Wind Products as Perceived by a Weather-Salient Sample. Journal of Operational Meteorology. 2017; 05(09):103–20. https://doi.org/10.15191/nwajom.2017.0509
4. Verbos RI, Brownlee MTJ. The Weather Dependency Framework (WDF): A tool for assessing the weather dependency of outdoor recreation activities. Journal of Outdoor Recreation and Tourism. 2017; 18:88–99. https://doi.org/10.1016/j.jort.2017.02.005
5. Scott D, Lemieux C. Weather and Climate Information for Tourism. Procedia Environmental Sciences. 2010; 1:146–83. https://doi.org/10.1016/j.proenv.2010.09.011
6. Paudyal R, Stein TV, Kc B, Adams DC. Effects of weather factors on recreation participation in a humid subtropical region. Int J Biometeorol. 2019; 63(8):1025–38. Epub 2019/04/11. https://doi.org/10.1007/s00484-019-01715-z PMID: 30969374.
7. Finger R, Lehmann N. Modeling the sensitivity of outdoor recreation activities to climate change. Climate Research. 2012; 51(3):229–36. https://doi.org/10.3354/cr01079
8. Matthews L, Scott D, Andrey J, Mahon R, Trotman A, Burrowes R, et al. Developing climate services for Caribbean tourism: a comparative analysis of climate push and pull influences using climate indices. Current Issues in Tourism. 2020; 24(11):1576–94. https://doi.org/10.1080/13683500.2020.1816928
9. Tucker P, Gilliland J. The effect of season and weather on physical activity: a systematic review. Public Health. 2007; 121(12):909–22. Epub 2007/10/09. https://doi.org/10.1016/j.puhe.2007.04.009 PMID: 17920646.
10. Baranowski T, Thompson WO, DuRant RH, Baranowski J, Puhl J. Observations on physical activity in physical locations: age, gender, ethnicity, and month effects. Res Q Exerc Sport. 1993; 64(2):127–33. Epub 1993/06/01. https://doi.org/10.1080/02701367.1993.10608789 PMID: 8341835.
11. Recreational Boating & Fishing Foundation. Outdoor Recreation Participation Report. 2018.
12. Allen T, Olds E, Southwick R, Scuderi B, Caputo L, Howlett D. Sportfishing in America: An economic force for conservation. American Sportfishing Association via Multistate Grant #F17AP00082 awarded by the Wildlife and Sport Fish Restoration Programs of the U.S. Fish and Wildlife Service, 2018.
13. Malvestuto SP, Davies WD, Shelton WL. Predicting the Precision of Creel Survey Estimates of Fishing Effort by use of Climatic Variables. Transactions of the American Fisheries Society. 1979; 108(1):43–5. https://doi.org/10.1577/1548-8659(1979)108<43:Ptpocs>2.0.Co;2
14. Graefe AR, Fedler AJ. Situational and subjective determinants of satisfaction in marine recreational fishing. Leisure Sciences. 1986; 8(3):275–95. https://doi.org/10.1080/01490408609513076
15. Griffin KJ, Hedge LH, Warton DI, Astles KL, Johnston EL. Modeling recreational fishing intensity in a complex urbanised estuary. J Environ Manage. 2021; 279:111529. Epub 2020/11/29. https://doi.org/10.1016/j.jenvman.2020.111529 PMID: 33246754.
16. Hunt LM, Boots BN, Boxall PC. Predicting Fishing Participation and Site Choice While Accounting for Spatial Substitution, Trip Timing, and Trip Context. North American Journal of Fisheries Management. 2007; 27(3):832–47. https://doi.org/10.1577/m06-079.1
17. Labriji A, El Foutayeni Y, Rachik M. Impact of wind speed on optimal fishing effort. Modeling Earth Systems and Environment. 2022. https://doi.org/10.1007/s40808-022-01360-3

18. Dundas SJ, von Haefen RH. The Effects of Weather on Recreational Fishing Demand and Adaptation: Implications for a Changing Climate. Journal of the Association of Environmental and Resource Economists. 2020; 7(2):209–42. https://doi.org/10.1086/706343

19. Administration) NNOaA. Marine Recreational Information Program. 2022. https://www.fisheries.noaa.gov/about/office-science-and-technology.

20. Obradovich N, Fowler JH. Climate change may alter human physical activity patterns. Nature Human Behaviour. 2017; 1(5). https://doi.org/10.1038/s41562-017-0097

21. Miller PW, Kumar A, Mote TL, Moraes FDS, Mishra DR. Persistent Hydrological Consequences of Hurricane Maria in Puerto Rico. Geophysical Research Letters. 2019; 46(3):1413–22. https://doi.org/10.1029/2018gl081591

22. Montane MM, Austin HM. Effects of Hurricanes on Atlantic Croaker (*Micropogonias undulatus*) Recruitment to Chesapeake Bay. In: Sellner KG, editor. Hurricane Isabel in Perspective: Proceedings of a conference. Edgewater, Maryland: Chesapeake Research Consortium, Inc.; 2005.

23. Valenti JL, Grothues TM, Able KW. Juvenile fish assemblage recruitment dynamics in a mid-Atlantic estuary: before and after Hurricane Sandy. Marine Ecology Progress Series. 2020; 641:177–93. https://doi.org/10.3354/meps13309

24. Greenwood MFD, Stevens PW, Matheson RE. Effects of the 2004 hurricanes on the fish assemblages in two proximate southwest Florida estuaries: Change in the context of interannual variability. Estuaries and Coasts. 2006; 29(6):985–96. https://doi.org/10.1007/bf02798660

25. Perret AJ, Kaller MD, Kelso WE, Rutherford DA. Effects of Hurricanes Katrina and Rita on Sport Fish Community Abundance in the Eastern Atchafalaya River Basin, Louisiana. North American Journal of Fisheries Management. 2010; 30(2):511–7. https://doi.org/10.1577/m09-065.1

26. Pita P, Ainsworth GB, Alba B, Anderson AB, Antelo M, Alós J, et al. First Assessment of the Impacts of the COVID-19 Pandemic on Global Marine Recreational Fisheries. Frontiers in Marine Science. 2021; 8. https://doi.org/10.3389/fmars.2021.735741

27. Apriesnig JL, Thompson JM. Recreational Marine Fishing in the Time of Covid-19. Agricultural & Applied Economics Association; Austin, TX2021.

28. Midway SR, Lynch AJ, Peoples BK, Dance M, Caffey R. COVID-19 influences on US recreational angler behavior. PLoS One. 2021; 16(8):e0254652. Epub 2021/08/19. https://doi.org/10.1371/journal.pone.0254652 PMID: 34407076

29. Keim BD, Powell EJ. Trends in Daily Temperature and Precipitation Extremes for the Southeastern United States: 1948–2012. Journal of Climate. 2015; 28(4):1592–612. https://doi.org/10.1175/jcli-d-14-00410.1

30. Miller PW, Trepanier JC. Predicting the Gulf of Mexico Hurricane Season With 500-hPa Temperature. Geophysical Research Letters. 2021; 48(17). https://doi.org/10.1029/2021gl094741

31. Coronavirus (Covid-19) Data in the United States [Internet]. 2021 [cited 2022-06-01]. https://github.com/nytimes/covid-19-data.

32. Fisheries LDoWa. Saltwater Finfish Landing Statistics. 2022.

33. Smith DR, Midway SR, Caffey RH, Penn JM. Economic values of Potential Regulation Changes for the Southern Flounder Fishery in Louisiana. Marine and Coastal Fisheries. 2022; 14(2):e10195. https://doi.org/10.1002/mcf2.10195

34. Hersbach H, Bell B, Berrisford P, Hirahara S, Horányi A, Muñoz-Sabater J, et al. The ERA5 global reanalysis. Quarterly Journal of the Royal Meteorological Society. 2020; 146(730):1999–2049. https://doi.org/10.1002/qj.3803

35. Gelman A, Hill J. Data Analysis Using Regression and Multilevel/Hierarchical Models: Cambridge University Press; 2006.

36. Plummer M. rjags: Bayesian Graphical Models using MCMC. http://mcmc-jags.sourceforge.net2016.

37. R Core Team. R: A language and environment for statistical computing. Vienna, Austria: R Foundation for Statistical Computing; 2022.

38. Knapp KR, Diamond HJ, Kossin JP, Kruk MC, Schreck CJI. International Best Track Archive for Climate Stewardship (IBTrACS) Project, Version 4. In: Information NNCfE, editor. 2018.

39. Britton JR, Pinder AC, Alos J, Arlinghaus R, Danylchuk AJ, Edwards W, et al. Global responses to the COVID-19 pandemic by recreational anglers: considerations for developing more resilient and sustainable fisheries. Reviews in Fish Biology and Fisheries. 2023:1–17. Epub 2023/06/26. https://doi.org/10.1007/s11160-023-09784-5 PMID: 37360579
